# Genome-wide microsatellite characteristics of five human *Plasmodium species*, focusing on *Plasmodium malariae* and *P. ovale curtisi*

**DOI:** 10.1051/parasite/2020034

**Published:** 2020-05-15

**Authors:** Vivek Bhakta Mathema, Supatchara Nakeesathit, Nicholas J. White, Arjen M. Dondorp, Mallika Imwong

**Affiliations:** 1 Department of Molecular Tropical Medicine and Genetics, Faculty of Tropical Medicine, Mahidol University 10400 Bangkok Thailand; 2 Mahidol–Oxford Tropical Medicine Research Unit, Faculty of Tropical Medicine, Mahidol University 10400 Bangkok Thailand; 3 Centre for Tropical Medicine and Global Health, Nuffield Department of Medicine, University of Oxford OX1 2JD Oxford United Kingdom

**Keywords:** Tandem repeats, Gene ontology, Microsatellite markers, Malaria

## Abstract

Microsatellites can be utilized to explore genotypes, population structure, and other genomic features of eukaryotes*.* Systematic characterization of microsatellites has not been a focus for several species of *Plasmodium*, including *P. malariae and P. ovale,* as the majority of malaria elimination programs are focused on *P. falciparum* and to a lesser extent *P. vivax.* Here, five human malaria species (*P. falciparum*, *P. vivax*, *P. malariae*, *P. ovale curtisi*, and *P. knowlesi*) were investigated with the aim of conducting in-depth categorization of microsatellites for *P. malariae* and *P. ovale curtisi*. Investigation of reference genomes for microsatellites with unit motifs of 1–10 base pairs indicates high diversity among the five *Plasmodium* species. *Plasmodium malariae,* with the largest genome size, displays the second highest microsatellite density (1421 No./Mbp; 5% coverage) next to *P. falciparum* (3634 No./Mbp; 12% coverage). The lowest microsatellite density was observed in *P. vivax* (773 No./Mbp; 2% coverage). A, AT, and AAT are the most commonly repeated motifs in the *Plasmodium* species. For *P. malariae* and *P. ovale curtisi*, microsatellite-related sequences are observed in approximately 18–29% of coding sequences (CDS). Lysine, asparagine, and glutamic acids are most frequently coded by microsatellite-related CDS. The majority of these CDS could be related to the gene ontology terms “cell parts,” “binding,” “developmental processes,” and “metabolic processes.” The present study provides a comprehensive overview of microsatellite distribution and can assist in the planning and development of potentially useful genetic tools for further investigation of *P. malariae* and *P. ovale curtisi* epidemiology.

## Abstract (fr)

Les microsatellites peuvent être utilisés pour explorer les génotypes, la structure de la population et d’autres caractéristiques génomiques des eucaryotes. La caractérisation systématique des microsatellites n’a pas été étudiée pour plusieurs espèces de *Plasmodium*, dont *P. malariae* et *P. ovale*, car la majorité des programmes d’élimination du paludisme se concentrent sur *P. falciparum* et, dans une moindre mesure, *P. vivax*. Dans cet article, cinq espèces causant le paludisme humain (*P. falciparum*, *P. vivax*, *P. malariae*, *P. ovale curtisi* et *P. knowlesi*) ont été étudiées dans le but de procéder à une catégorisation approfondie des microsatellites pour *P. malariae* et *P. ovale curtisi*. L’étude des génomes de référence pour les microsatellites avec des motifs unitaires de 1 à 10 paires de bases indique une grande diversité parmi les cinq espèces de *Plasmodium*. *Plasmodium malariae*, avec la plus grande taille de génome, affiche la deuxième densité de microsatellites la plus élevée (1421 No./Mpb ; 5 % de couverture) après *P. falciparum* (3634 No./Mpb ; 12 % de couverture). La plus faible densité de microsatellites a été observée chez *P. vivax* (773 No./Mpb ; 2 % de couverture). A, AT et AAT sont les motifs les plus fréquemment répétés chez les espèces de *Plasmodium*. Pour *P. malariae* et *P. ovale curtisi*, des séquences liées aux microsatellites sont observées dans environ 18 à 29 % des séquences codantes (CDS). La lysine, l’asparagine et les acides glutamiques sont les plus souvent codés par les CDS liés aux microsatellites. La majorité de ces CDS pourrait être liée aux termes d’ontologie génétique « parties cellulaires », « liaison », « processus de développement » et « processus métaboliques ». Cette étude fournit un aperçu complet de la distribution des microsatellites et peut aider à la planification et au développement d’outils génétiques potentiellement utiles pour une étude plus approfondie de l’épidémiologie de *P. malariae* et *P. ovale curtisi*.

## Introduction

Recent advancements in gene sequencing technologies and the increasing availability of online genomic resources have made it possible to computationally explore genomic features of an organism that were previously inaccessible [18]. Molecular genetics and polymorphism studies involving microsatellites are among the key beneficiaries of such technological advancement. Microsatellites are short tandem repeats of DNA usually consisting of 1–10 base pair (bp) unit nucleotide motifs. Such microsatellites are known to be formed due to mispairing, improper alignment, and strand-slippage events [13, 22, 25]. Microsatellites with unit motifs of 2–3 bp are often designated as short tandem repeats, simple sequence repeats, and simple sequence length polymorphisms [44, 64]. Moreover, these microsatellites can be characterized as (i) perfect repeats containing only pure motifs with 100% identical copies and constituting only one motif type; (ii) imperfect repeats containing motifs with mutations such as insertions, deletions, or substitution; and (iii) compound microsatellites containing stretches of two or more different repeat motifs [8]. These short tandem repeats of DNA can be highly polymorphic and are widely distributed throughout the genome of eukaryotic cells. Microsatellites are usually abundant in non-coding regions of the genome and can be targeted to produce polymerase chain reaction (PCR) products as markers to identify genetic diversity among a population [13, 25]. Currently, microsatellite markers are implemented across wide fields of biology, including gene linkage, genotyping, forensics, kinship relationships, phylogenetic analysis, and others [54]. Conventional procedures for microsatellite studies consist of *in vitro* microsatellite motif cloning, which is screening of cloned libraries for restricted motif types with often limited prior knowledge of microsatellite categorization and distribution. Such protocols are expensive, time-consuming, suffer from low modularity, and are prone to experimental errors. In contrast, recent years have witnessed a stark increase in the use of *in silico* tools for the analysis of microsatellites utilizing publicly available genomic databases [55]. Moreover, entire online platforms dedicated to particular groups of organisms (e.g., PlasmoDb and VivaxGen) and genome projects are significantly enhancing the range and accuracy of *in silico* analysis approaches [4, 62].

Decades long malaria intervention strategies have significantly reduced the number of malaria cases and fatalities worldwide. The Greater Mekong Subregion (GMS) has achieved significant progress in reducing the disease burden to meet their target of malaria elimination by 2015–2030. The GMS countries have achieved a 54% reduction in the incidence of malaria cases between 2012 and 2015 and the death rate has fallen by 84% over the same period [66]. The actual drug efficacy and burden of non-*falciparum* malaria still remain unclear due to the lack of sufficient epidemiological tools to investigate these parasite variants.

Instead of targeting all *Plasmodium* species, most malaria elimination programs are predominantly directed toward *P. falciparum* and to a lesser extent *P. vivax* [33]. Non-*P. falciparum* malaria, mainly by *P. malariae* and *P. ovale,* still presents a major challenge for malaria eradication [32, 68]. *Plasmodium malariae* infects humans and causes fever. These infections are usually asymptomatic with low parasitemia but may cause chronic anemia and nephrotic syndrome [12, 17, 29, 41]. *Plasmodium ovale* can be subcategorized into two distinct species, *P. ovale curtisi* and *P. ovale wallikeri*, which only differ by small genetic variations and a shorter latency period in *P. ovale wallikeri* [39]. These sympatric-occurring *P. ovale* subspecies are generally indistinguishable morphologically. Infections by either of these *P. ovale* subspecies present with mild fever and are currently treated with the conventional antimalarial drug chloroquine [40]. *Plasmodium ovale* can undergo the hypnozoite stage, which is a dormant stage in the liver. This enables concealment from diagnosis, and reactivation may occur weeks, months, or even years after the initial infection, leading to disease relapse [21, 48]. This parasite is endemic throughout parts of Asia, Africa, South America, and the Western Pacific [29, 43, 50, 69].

Recent epidemiological studies conducted in Cameroon [47] and Equatorial Guinea [53] have revealed the presence of over 12% *P. malariae* followed by 1–6% *P. ovale-*positive samples in parts of Uganda and Bioko [43]. Separate studies conducted in Tanzania indicate persistent transmission of *P. malariae* and *P. ovale* in an area of declining *P. falciparum* [68]. These findings collectively signal the significant presence of these parasites and reveal an epidemiologic knowledge gap between them and other well-studied *Plasmodium* species [51].

Barely adequate genetic markers available for *P. malariae* and *P. ovale curtisi* compared to *P. falciparum* [2, 16, 20, 57] and *P. vivax* [19, 24, 37] self-elaborate the low emphasis being given to these parasites. Microsatellite-based schemes would greatly facilitate population genetics and therapeutic studies in *P. malariae* and *P. ovale curtisi*. Large genome size (~29 Mbp) with high AT content (~75%) ideally make microsatellite-based genotyping markers a suitable means for investigating epidemiology and population genetics of these *Plasmodium* species [4]. This study aims to present comprehensive categorization of the microsatellite distribution of major human malaria-causing *Plasmodium* species with a focus on *P. malariae* and *P. ovale curtisi,* which may also contribute to the development of additional genotyping markers for this parasite.

## Materials and methods

### Sequencing data

This study is a review and bioinformatics analysis of microsatellites in five human malaria-causing *Plasmodium* species based on whole genome sequencing data available in the PlasmoDB database. The whole genome sequences of *P. malariae* UG01, *P. falciparum* 3D7*, P. vivax* SAL-1, *P. ovale curtisi* GH01, and *P. knowlesi* STRAIN-H were downloaded from the PlasmoDB webserver (http://plasmodb.org/common/downloads/release-36/) [4]. *Plasmodium ovale wallikeri* was not included in the analysis due to its close genetic relatedness to *P. ovale curtisi* and lack of a standardized reference genome in the PlasmoDB database [4, 43]. Nucleotide sequences of all predicted and known coding sequence (CDS) regions for each *Plasmodium species* were obtained using the PlasmoDB webserver’s built-in gene resource download tools. The *Plasmodium* strains with maximum known genes and transcripts in the PlasmoDB webserver were selected for each species under evaluation. The total number of nucleotide base pairs scanned for microsatellites and whole genome GC% content of each organism are listed in Table 1. For *P. malariae* and *P. ovale curtisi*, sets of 6573 and 7162 sequences representing ≥98% of the total available CDS (known and predicted proteins) from the whole-genome sequence were included for evaluation.

**Table 1:** Genome-wide coverage and density of microsatellites in the genomes of five *Plasmodium* species.

Type	Microsatellite features	*P. falciparum* 3D7	*P. vivax* SAL-1	*P. malariae* UG01	*P. ovale curtisi* GH01	*P. knowlesi* STRAIN-H
Genome-wide microsatellites	Sequence analyzed (bp)	23,332,839	27,013,980	33,618,035	33,479,509	24,395,979
	Genomic GC content (%)	19.34	42.19	24.38	28.48	38.60
	No. of microsatellites	84,786	20,875	47,762	29,245	32,008
	Microsatellite density (No./Mbp)	3633.76	772.75	1420.73	873.52	1312.02
	Microsatellite occurrence per 2 Kb	7.30	1.55	2.84	1.75	2.62
	Total length of microsatellite (bp)	2,698,077	591,020	1,663,135	793,213	1,129,172
	Microsatellite coverage (bp/Mbp)	115,634.32	21,878.30	49,471.51	23,692.49	46,285.17
	Perfect microsatellites (%)	56.09	70.22	61.71	76.70	71.32
	Genome content by microsatellite (%)	11.56	2.19	4.95	2.37	4.63
CDS microsatellites	No. of microsatellites	9284	1766	4107	2364	1447
	Microsatellite density (No./Mbp)	397.89	65.37	122.17	70.61	59.31
	Total length of microsatellites (bp)	367,916	59,733	184,537	83,447	43,055
	Microsatellite coverage (bp/Mbp)	15,768.16	2211.19	5489.23	2492.48	1764.84
	Perfect microsatellites (%)	47.73	50.23	48.31	56.98	59.23

### Microsatellite analysis

Identification and categorization of perfect and imperfect microsatellites was performed with the highly accurate tandem repeat search tool Phobos version 3.3.11 (http://www.ruhr-uni-bochum.de/ecoevo/cm/cm_phobos.htm) [35, 56]. The total GC% content and basic genomic statistics for each parasite sample were calculated with the python script multifastats.py (https://github.com/davidrequena/multifastats/blob/master/multifastats.py). The detection criteria for tandem repeats was restricted to evaluation of perfect and imperfect repeats with unit motifs of 1–10 bp with a minimum threshold repeat number of 14, 7, 5, 4, 4, 4, 4, 4, 4, and 4 for mono-, di-, tri-, tetra-, penta-, hexa-, hepta-, octa-, nona-, and deca-nucleotide microsatellites, respectively. For protein sequences, microsatellite GC content and tandemly repeated residues with a minimum of four repeats and maximum unit motif length of three amino acids were considered for evaluation using OSTRFPD [34]. Analysis of tandemly repeated amino acid sequences for *P. malariae* and *P. ovale curtisi* included the entire set of CDS available in the PlasmoDB online database.

### Heatmaps and genomic visualization of microsatellites

Clusters of microsatellites present in CDS regions of each chromosome were visualized as heatmaps using the seaborn library-backend python script (https://seaborn.pydata.org) with Euclidean metrics and complete linkage as measurement parameters. Scatter plots with Spearman’s correlation coefficients were generated to compute correlations between unit motif length and frequency of microsatellites using the seaborn library-backend python script. Statistical significance was defined as *p*-values < 0.05. Circos version 0.67-7 (http://circos.ca/) was used to visualize the genome-wide distribution of microsatellites.

### Gene ontology analysis

The Gene ontology (GO) terms associated with the cellular components, molecular functions, and biological processes for the microsatellite-associated protein sequences were computed by the deep neural-net-based hierarchical biological sequence classifier “SECLAF” using its default parameters trained on the UniProtKB GO database [58]. Microsatellite-associated proteins with the highest values of predicted GO terms each exceeding the 0.95 threshold score were included for analysis.

## Results

### Abundance, distribution, and diversity of microsatellites in five *Plasmodium* species

The analysis was conducted for both perfect and imperfect microsatellites with repeat numbers of 14, 7, 5, 4, 4, 4, 4, 4, 4, and 4 for 1–10 bp unit motif lengths, respectively, to minimize selection of nominal functional repeats related to the extreme AT-richness of some *Plasmodium* genomes. The genome-wide numbers of microsatellites identified among the five *Plasmodium* species were highly variable (84,786–20,875), along with the genomic coverage ranging from 11.56% to 2.19% (Table 1). The largest difference in both microsatellite density and coverage was observed between *P. falciparum* and *P. vivax* with densities of 3269.46 and 627.05 microsatellites per million base pairs (No./Mbp) and coverage of 115,634.32 and 21,878.30 microsatellite repeats per million base pairs (bp/Mbp)*,* respectively. *Plasmodium vivax* exhibited the lowest microsatellite density (772.75 No./Mbp) and coverage (21,878.30 bp/Mbp). The average number of microsatellite occurrences for *P. falciparum*, *P. vivax, P. malariae*, and *P. ovale* were 7.3, 1.55, 2.84, and 1.75 microsatellites per 2 kilobase pair (kbp) genome length, respectively. *Plasmodium malariae* with the largest genome size harbored 47,762 microsatellites with a microsatellite density of 1420.73 No./Mbp, the second highest among all species investigated (Table 1). The highest and lowest percentages of perfect microsatellites were observed in *P. ovale curtisi* (76.70%) and *P. falciparum* (56.09%), respectively (Table 1). The maximum (76.70%) and minimum (56.09%) percentages of perfect microsatellites were observed in *P. ovale curtisi* and *P. falciparum,* respectively (Table 1)*.* The highest density (397.90 No./Mbp) and coverage (15,768.16 bp/Mbp) of CDS-associated microsatellites were observed in *P. falciparum*; the lowest density (65.37 No./Mbp) and coverage (2211.19 bp/Mbp) were observed in *P. vivax* (Table 1). Microsatellite density in *P. malariae* was 1.63-fold higher than that in *P. ovale curtisi* (Table 1)*.*

**Figure 1:**
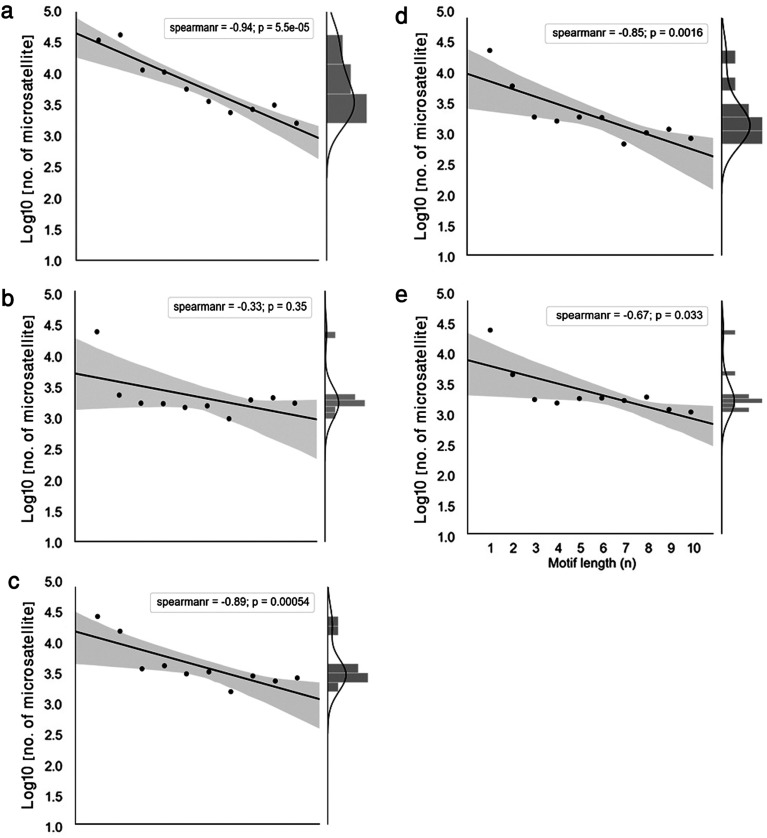
Unit motif length and frequency of microsatellites in seven *Plasmodium* species. Distribution of microsatellites in five species of *Plasmodium* expressed as percentage of motif length for (a) *P. falciparum* 3D7, (b) *P. vivax* SAL-1, (c) *P. malariae* UG01, (d) *P. ovale curtisi* GH01, and (e) *P. knowlesi* STRAIN-H. Trend line indicates logarithmic decline in number of microsatellites with increase in motif length. Histograms associated with each scatter plot represent kernel density estimates for frequency of unit motif type.

There was a high degree of diversity among the *Plasmodium* species in unit motif lengths of microsatellites across the respective genomes (Table 2). *Plasmodium falciparum* had the highest numbers and densities of mono- to penta-nucleotide long motif repeats, whereas *P. vivax* showed the lowest (Table 2). *Plasmodium malariae* showed the highest number of repeats for hexa-, octa-, and deca-nucleotide long motif repeats totaling 1648, 1353, and 1221, respectively. However, because of its large genome size, this did not translate to the highest repeat density or coverage of these motifs (Table 2). In general, the mono-, di-, and tri-nucleotide motifs were most abundant and collectively they accounted for approximately 80.0–90.0% of the total genome-wide unit motif length in all *Plasmodium* species. The highest proportions of mono-, di-, tri-, and tetra-nucleotides were observed in *P. vivax* (74.46%), *P. falciparum* (41.78%), *P. falciparum* (8.19%), and *P. falciparum* (7.34%), respectively (Table 2). *Plasmodium malariae* was found to harbor the highest percentage of hexa-nucleotide long motifs (3.45%) with a corresponding microsatellite density of 49.02 No./Mbp (Table 2). Interestingly, *P. malariae* had 3-fold higher microsatellite density compared to that of *P. ovale curtisi* for the unit motif lengths two, four, seven, and nine (Table 2). Nonetheless, *P. falciparum, P. malariae,* and *P. ovale* showed a clear negative correlation between 1 bp and 10 bp unit motif lengths and the frequency of microsatellite occurrence (Spearman’s *R* ≤ −0.85, *p* ≤ 1.6e-03). The negative correlation was also present for *P. vivax* (Spearman’s *R* = −0.33, *p* = 0.35) and *P. knowlesi* (Spearman’s *R* = −0.67, *p* = 0.033), albeit to a weaker degree (Fig. 1).

**Table 2:** Relative density and categorization of 1-10 bp unit motif length microsatellites in the genomes of five *Plasmodium* species.

Unit motif length (bp)	Features	*P. falciparum* 3D7	*P. vivax* SAL-1	*P. malariae* UG01	*P. ovale curtisi* GH01	*P. knowlesi* STRAIN-H
Mononucleotide (*n* = 1)	No.	27,687	15,544	24,320	20,985	23,807
	Percentage (%)^a^	32.66	74.46	50.919	71.756	74.378
	No./Mbp	1186.61	575.40	723.42	626.80	975.86
	Length coverage	644,936	317,981	506,974	485,595	567,513
	Coverage/Mbp	27,640.70	11,770.98	15,080.42	14,504.24	23,262.56
Dinucleotide (*n* = 2)	No.	35,423	846	11,857	3721	2642
	Percentage (%)^a^	41.779	4.053	24.86	12.724	8.254
	No./Mbp	1518.16	31.38	352.69	111.14	108.30
	Length coverage	1,155,503	22,208	350,048	86,288	85,158
	Coverage/Mbp	49,522.61	822.09	10,412.51	2577.34	3490.66
Trinucleotide (*n* = 3)	No.	6944	595	1911	840	772
	Percentage (%)^a^	8.19	2.85	4.001	2.872	2.412
	No./Mbp	297.61	22.03	56.84	25.09	31.64
	Length coverage	243,265	15,766	84,872	25,951	21,436
	Coverage/Mbp	10,425.87	583.62	2524.60	775.13	878.67
Tetranucleotide (*n* = 4)	No.	6228	575	2247	686	646
	Percentage (%)^a^	7.346	2.754	4.705	2.346	2.018
	No./Mbp	266.90	21.28	66.90	20.49	26.48
	Length coverage	172,622	18,218	120,340	26,431	23,432
	Coverage/Mbp	7398.24	674.39	3579.63	789.47	960.49
Pentanucleotide (*n* = 5)	No.	2838	486	1510	841	809
	Percentage (%)^a^	3.347	2.328	3.162	2.88	2.527
	No./Mbp	121.63	17.99	44.92	25.12	33.16
	Length coverage	98,990	16,494	65,863	27,158	34,798
	Coverage/Mbp	4242.52	610.57	1959.16	811.18	1426.38
Hexanucleotide (*n* = 6)	No.	1635	523	1648	803	830
	Percentage (%)^a^	1.928	2.505	3.45	2.746	2.593
	No./Mbp	70.07	19.36	49.02	23.98	34.02
	Length coverage	77,483	22,263	111,744	39,628	43,860
	Coverage/Mbp	3320.77	824.13	3323.93	1183.65	1797.84
Heptanucloetide (*n* = 7)	No.	955	289	629	225	742
	Percentage (%)^a^	1.126	1.384	1.317	.769	2.318
	No./Mbp	40.93	10.70	18.71	6.72	30.41
	Length coverage	78,852	50,031	53,547	15,988	217,069
	Coverage/Mbp	3379.44	1852.04	1592.81	477.56	8897.74
Octanucleotide (*n* = 8)	No.	1123	681	1353	383	872
	Percentage (%)^a^	1.325	3.262	2.833	1.31	2.724
	No./Mbp	48.13	25.21	40.25	11.44	35.74
	Length coverage	65,740	39,529	124,078	25,271	68,224
	Coverage/Mbp	2817.49	1463.28	3690.82	754.82	2796.52
Nonanucleotide (*n* = 9)	No.	1367	749	1066	465	474
	Percentage (%)^a^	1.612	3.588	2.232	1.59	1.481
	No./Mbp	58.59	27.73	31.71	13.89	19.43
	Length coverage	102,348	46,073	108,343	37,044	36,635
	Coverage/Mbp	4386.44	1705.52	3222.77	1106.47	1501.68
Decanucleotide (*n* = 10)	No.	586	587	1221	296	414
	Percentage (%)^a^	0.691	2.812	2.556	1.012	1.293
	No./Mbp	25.10	21.73	36.319	8.84	16.97
	Length coverage	41,816	44,108	137,797	24,354	32,881
	Coverage/Mbp	1792.15	1632.78	4098.90	727.43	1347.80

a Number of microsatellites of unit motif length (*n*)/total number of microsatellites.

### A, AT, and AAT as the most dominant microsatellite motifs in *Plasmodium* genomes

The microsatellite motif sequences show high diversity among the different *Plasmodium* species (Supplementary Table 1), although motif type A was repeated most frequently in all species of *Plasmodium* except *P. falciparum,* where AT (41.62%) was the most common repeat. In contrast, AG was the least frequently occurring di-nucleotide motif in all *Plasmodium* species under investigation (Supplementary Table 1). The motifs A, AT, and AAT collectively accounted for more than 70% of all repeats in the studied species. Motifs containing only C and G were relatively rare (<10%) for mono- and di-nucleotide repeats. Only *P. vivax* harbored frequently repeated tri-nucleotide motifs AGG with a microsatellite GC content > 50%. In *P. malariae*, the number of mono-nucleotide motif repeats A was more than twice the number of di-nucleotide AT motif repeats (Supplementary Table 2).

**Figure 2:**
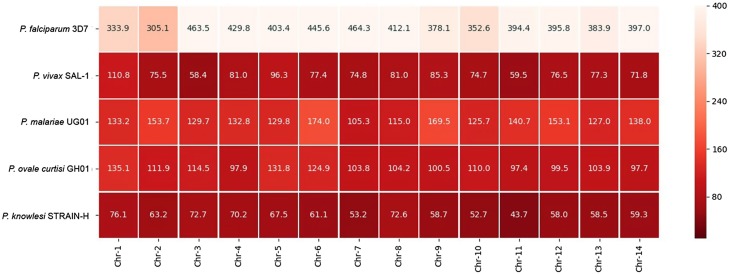
Microsatellite distribution in coding regions of seven *Plasmodium* species. Heatmap indicating microsatellites expressed as chromosomal density identified in coding regions (CDS) of five *Plasmodium* species. Heatmap color key indicates microsatellite density (No./Mbp). Chr, chromosome.

The CDS microsatellite density distribution in protein-coding regions of *Plasmodium* analyzed for individual chromosomes was computed as heatmaps (Fig. 2). The highest microsatellite density was on chromosome 6 of *P. falciparum* (464.30 No./Mbp), and the lowest density was on chromosome 11 of *P. knowlesi* (43.70 No./Mbp). Heatmap analysis showed that *P. falciparum* (397.12 No./Mbp)*, P. malariae* (137.67 No./Mbp), and *P. ovale* (109.50 No./Mbp) had an average CDS-associated chromosomal microsatellite density greater than 100 No./Mbp, whereas *P. vivax* (78.58 No./Mbp) and *P. knowlesi* (61.95 No./Mbp) showed average microsatellite densities of less than 100 No./Mbp (Fig. 2).

### Diversity of tandemly repeated amino acids in microsatellite-associated gene products of *P. malariae* and *P. ovale curtisi* and their ontology annotations

**Figure 3:**
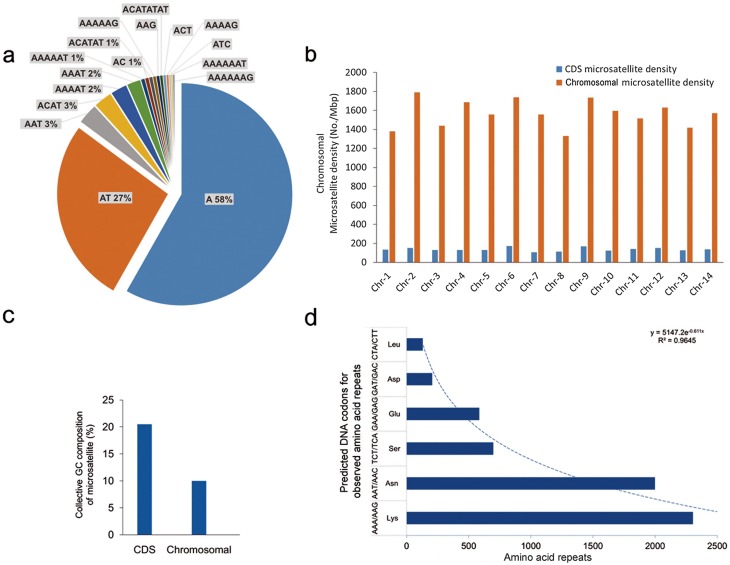
Microsatellite motif, chromosomal density, and tandemly repeated amino acid residue in *Plasmodium malariae* UG01. Representative charts for (a) 10 most frequently repeated motifs in chromosomes, (b) comparative distribution of total microsatellites in total chromosomal and coding sequences (CDS) expressed as microsatellite density, (c) aggregate GC content of microsatellites in CDS and total chromosomal sequences, and (d) six most frequent tandemly repeated amino acids associated with microsatellites. Trend line indicates logarithmic decline in number of tandemly repeated amino acids.

**Figure 4:**
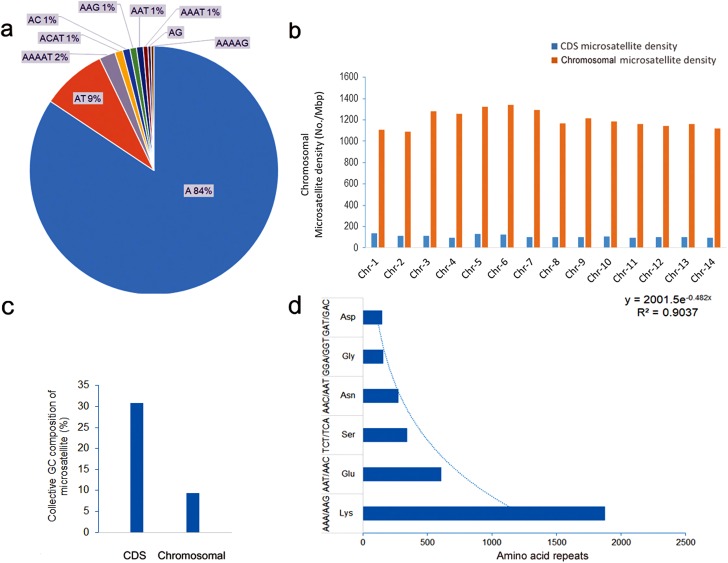
Microsatellite motif, chromosomal density and tandemly repeated amino acid residue in *Plasmodium ovale curtisi* GH01. Representative charts for (a)10 most frequently repeated motifs in chromosomes, (b) comparative distribution of total microsatellites in total chromosomal and coding sequences (CDS) expressed as microsatellite density, (c) aggregate GC content of microsatellites in CDS and total chromosomal sequences, and (d) six most frequent tandemly repeated amino acids associated with microsatellites. Trend line indicates logarithmic decline in number of tandemly repeated amino acids.

Motif-wise distributions of microsatellites in the *P. malariae* and *P. ovale curtisi* chromosomes were investigated further. The mono-, di-, and tri-nucleotide motifs accounted on average for 50, 25, and 5% in *P. malariae* and 75, 9, and 2% in *P. ovale curtisi* of total unit motif repeats, respectively (Supplementary Table 2). The collective contribution of tri- to deca-nucleotide motifs was ≤10% of the total repeats in both species (Supplementary Table 2). On average, the chromosomal microsatellite densities for *P. malariae* and *P ovale curtisi* were 1568.22 ± 140.48 and 1203.04 ± 82.28 No./Mbp, respectively. For *P. malariae*, the A, AT, and AAT unit repeat motifs were the most frequent, constituting 58, 28, and 3% of total chromosomal DNA microsatellites, respectively (Fig. 3a). The total microsatellite densities in non-coding regions were 7.87–10.01-fold higher than in the CDS regions (Fig. 3b). Aggregate GC content of CDS-associated microsatellites was approximately 2-fold higher (21.0%) compared to that in the chromosomal region (Fig. 3c). Evaluation of the amino acid repeats in the annotated and predicted proteins available for *P. malariae* showed that lysine (34.12%) and asparagine (29.58%) were the most common amino acid repeats, corresponding to the most commonly observed tri-nucleotide repeats AAA (61%) and AAT (4%) (Fig. 3a and d). For *P. ovale curtisi,* the A, AT, and AAAAT repeat motifs were most frequent in aggregate chromosomal DNA, constituting 84%, 9%, and 2%, respectively (Fig. 4a). The microsatellite densities in non-coding regions were 7.77–12.20-fold higher than in CDS regions (Fig. 4b). Aggregate GC content of CDS-associated microsatellites was approximately 3-fold higher (30.0%) compared to that of the chromosomal region (Fig. 4c). Evaluation of the amino acid repeats indicated that lysine (34.12%) and asparagine (29.58%) were the most common amino acid repeats, corresponding to the most commonly observed tri-nucleotide repeats AAA (61%) and AAT (4%) (Fig. 4d).

**Figure 5:**
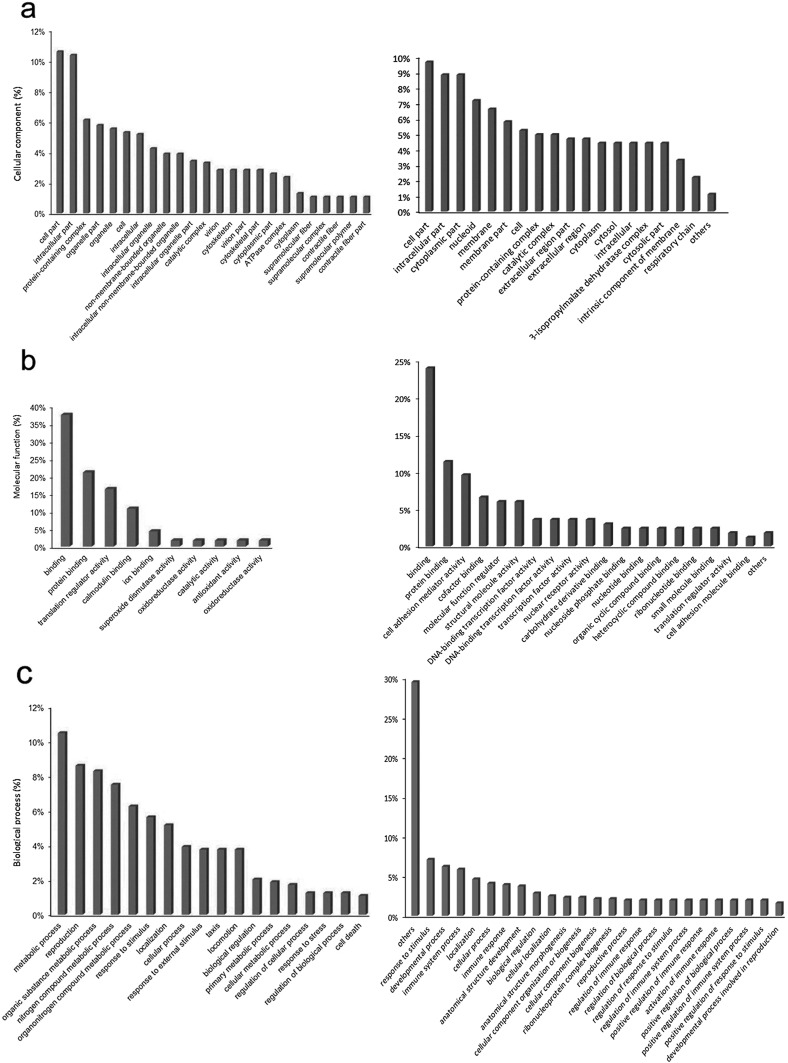
Gene ontology of microsatellites associated proteins in *Plasmodium malariae* UG01 (left) and *P. ovale curtisi* GH01 (right). Representative bar chart indicating the major gene ontologies of microsatellite-associated coding sequences (CDS) for (a) cellular component, (b) molecular function, and (c) biological process. Values for GO terms in the bar diagrams with less than 1.0–1.5% coverage are not indicated.

For investigating functional annotation of the microsatellite-associated CDS distribution, the SECLAF classifier was trained for over 980 GO classes with an area under the curve (AUC) of 99.45% [28], providing a rough estimate of GO for largely unclassified microsatellite-linked proteins of *P. malariae* and *P. ovale curtisi*. For *P. malariae* and *P. ovale curtisi*, only 2054 and 1555 sequences were assigned to specific gene names and descriptions, respectively. In total, 1919 and 1271 distinct CDS were found to contain at least one microsatellite for *P. malariae* and *P. ovale curtisi*, respectively. For *P. malariae*, three major GO categories, cellular component, molecular function, and biological process, were assigned to 229, 810, and 874 microsatellite-associated proteins. Within the categories, the top three GO terms collectively represent at least 25% of the total GO diversity. Regarding microsatellite-associated proteins under the “cellular component” GO category, the GO terms “cell parts,” “protein-containing complex,” and “intracellular part” collectively constituted over 40% of the total ontologies (Fig. 5a). The three major GO terms with regard to molecular function for microsatellite-associated proteins were “binding,” “protein binding,” and “translation regulatory activity” (Fig. 5b). The three major GO terms constituting “biological process” were “metabolic process,” “reproduction,” and “organic substance metabolic process” (Fig. 5c). For *P. ovale curtisi*, three major GO categories, cellular component, molecular function, and biological process, were assigned to 513, 146, and 446 microsatellite-associated proteins, respectively. In each category, the top three GO terms collectively represented at least 30% of the total GO diversity. GO categorized under “cellular component” displayed “cell parts,” “intracellular part,” and “cytoplasmic part” as the three major GO terms that collectively constituted over 40% of the total ontologies (Fig. 5a). The three major GO terms with regard to molecular function for microsatellite-associated proteins were “binding,” “protein binding,” and “cell adhesion mediator activity” (Fig. 5b). The three major GO terms constituting “biological process” were “response to stimulus,” “developmental process,” and “immune system process” (Fig. 5c).

### Microsatellite distribution map for *P. malariae* and *P. ovale curtisi*

**Figure 6:**
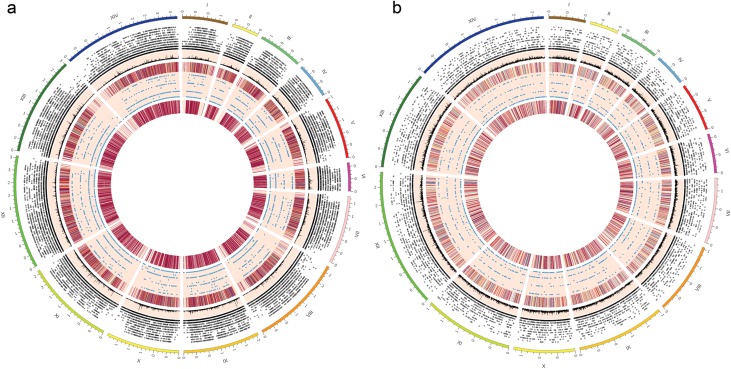
Genome-wide representation of microsatellite distribution map for *Plasmodium malariae* UG01 and *P. ovale curtisi* GH01. Different features indicated by microsatellite distribution map for (a) *P. malariae* UG01 and (b) *P. ovale curtisi* GH01 from outermost^a^ to innermost ring can be interpreted as: chromosome 1–14 (I–XIV), scatter plotb^b^ for genomic microsatellite distribution based on unit motif length which corresponds to the height of spot from base of its ring, line plot with peaks indicating regions with long repeat length, heatmap^c^ corresponding to the aggregate genomic microsatellite, scatter plot for microsatellites present in protein-coding region, heatmap for the aggregate microsatellites presenting in protein coding region of the genome. ^a^Each unit difference in outermost ring represents chromosomal length of 1 mega base pair. ^b,c^Spots and regions in scatter plot and heatmap may appear overlapped due to high density but are physically apart in sequence.

A graphical representation (Fig. 6) comprising the entire known chromosomal DNA of *P. malariae* and *P. ovale curtisi* shows a relatively homogeneous distribution of microsatellites on each chromosome. For *P. malariae* and *P. ovale,* the density of genomic microsatellites in non-CDS sequences is ≥10-fold greater than in CDS regions (Figs. 3b and 5). Most of the microsatellites with smaller unit motifs (<4 bp) were homogenously distributed, whereas microsatellites with longer unit motifs (>4 bp) appeared to be more concentrated toward the middle region of the chromosome (Fig. 6, Supplementary Figures 1 and 2). In general, 1–3 unit motif microsatellites had high densities (Supplementary Figure 3). The genome-wide microsatellites with longer repeats, which appeared as peaks in the line chart within the map, were found to be more frequent around the middle region of most chromosomes (Fig. 6).

## Discussion

In the absence of a standardized classification metric, the present study provides a comprehensive categorization and distribution of microsatellites in five human malaria-causing *Plasmodium* species. Strand slippage mutations, improper pairing, and host-parasite adaptation history may have contributed to the wide variation in microsatellite density and GC% content among the *Plasmodium* species. At present, aside from the distinct individual genomic nature of each *Plasmodium* species and wide variation in GC% content, there is no concrete evidence to justify or speculate on the observed variations in microsatellite motifs among the species. Previous studies have suggested microsatellites as a major contributor for genetic diversity in *P. falciparum* and *P. vivax* populations [45, 59, 61]. Such high densities of short tandem repeats not only increase nucleotide polymorphisms but also complicate genetic analysis [5, 59, 61]. Earlier studies without the availability of the highly accurate Phobos search tool have suggested that a microsatellite may occur for every 2–3 kbp in the *P. falciparum* genome [1]. The present survey of the *P. falciparum* genome shows comparatively high microsatellite density, totaling 3633.76 No./Mbp, which corresponds to approximately six microsatellites per 2 kbp genome length. The use of more sophisticated tools to evaluate perfect/imperfect microsatellites and the high quality of genome sequence might have contributed to the observed difference in current estimates. Microsatellites have been reported in over 9% of ORFs for the extremely AT-rich (~80%) *P. falciparum* genome, which is in agreement with the current observation [59]. Likewise, a similar trend was visible for *P. malariae* and *P. ovale curtisi*, indicating substantial microsatellite-associated heterozygosity and its potential exploitation to access genomic diversity. All *Plasmodium* species displayed a logarithmic decline in the frequency of microsatellites with increasing unit motif length. This is likely to be related to the mechanisms of DNA slippage and DNA mismatch repair, which result in a greater likelihood of generating shorter AT-rich simple motifs [14, 28, 59]. The estimated slippage mutation rate within microsatellites has been suggested to increase exponentially as the length of the repeat motif increases [28]. This phenomenon is reflected by the observed high percentage of short perfect 1–3 unit motif microsatellites (≥60%) in all species of *Plasmodium* under investigation. Thus, these factors result in a higher microsatellite mutation rate compared to the single point mutation rates. *Escherichia coli* [30], mice [10], humans [65], and *P. falciparum* [2] are reported to have microsatellite mutation rates of approximately 1 × 10^−2^, 10^−3^−10^−4^, 1 × 10^−3^, and 6.95 × 10^−5^–3.7 × 10^−4^/locus/replication, respectively, which are all higher than the single point mutation rates in these organisms. A lack of analysis for Hardy–Weinberg equilibrium and linkage disequilibrium are among the few limitations of the present study as only the standard whole genome sequence of each *Plasmodium* species was investigated. *Plasmodium malariae* and *P. ovale curtisi* have over 1000 known microsatellite-related CDS constituting at least 10% of the entire genome, which are mainly distributed across 14 chromosomes [4]. Microsatellite instability in these CDS could promote protein domain duplication and production of homo-peptide tracts and interfere with transcript splicing, leading to disorders and disease [42, 67]. However, natural selection tends to favor suppression of tandem repeats in CDS compared to that in non-coding regions [38, 60]. The extreme AT richness in *P. falciparum* has been reported to contribute to systematic mutational bias, resulting in abnormally high microstructural plasticity; thus, such studies have not yet been assessed for *P. malariae* [23]. Nonetheless, microsatellite-associated polymorphisms may facilitate the adaptability of *P. malariae* in primate hosts, including South American primates and chimpanzees [49].

AT-richness of the *P. malariae* and *P. ovale curtisi* genomes was in accordance with the high AT content (89%) of CDS-associated microsatellite sequences. Additionally, hydrophilic amino acids such as lysine and asparagine were among the most commonly repeated amino acid motifs observed, which is consistent with the natural bias towards hydrophilic amino acids in proteins [26]. Lysine-rich short tandemly repeated sequences have been observed in different protozoal parasites, including *P. falciparum* and *Leishmania major.* These parasites are suggested to generate such amino acid sequences *de novo* to modulate host protein targeting efficiency [11, 36]. Because microsatellite instability in CDS could increase the chance of forming mutant proteins, the study of GO terms for such CDS-associated proteins should not be ignored. Although GO analysis of microsatellite-related CDS has been fairly limited to UniProtKB-based interpretation of known protein sequences, an overview of annotation for cellular components, molecular functions, and biological processes may be useful for high-level interpretation of microsatellite-associated proteins. In *P. malariae* and *P. ovale curtisi*, the majority of ontological terms for proteins associated with microsatellite-containing CDS were assigned to “cell parts,” “binding,” “metabolic process,” and “response to stimuli” which are often linked to cellular integrity, adaptation, and survival of pathogens [3, 6, 15]. The relationship between microsatellite content and plasticity of these ontologies is an interesting area for further study.

An important aspect of the genome-wide microsatellite mapping in this study is to facilitate development of genotyping markers. Unlike SNP and DNA barcoding, microsatellite markers can be evaluated for the entire genome of an organism [7, 52]. Although SNPs in merozoite surface proteins (*msp*) 1 and *msp2* have been used to investigate polymorphisms in *Plasmodium* species, the pressure from the selective host immune system often reduces polymorphisms and subsequently lowers the application of such protein gene-based markers [9, 31]. In contrast, the majority of microsatellites are in non-coding regions that can achieve a high degree of polymorphism, making it a suitable marker for discriminating variants within a population and drug efficacy studies [27, 64]. Mass drug administration programs expose a parasite to antimalarials, which further suggests the urgency in investigating genetic diversity within *P. malariae* and *P. ovale curtisi* [63]. Although identification of genotyping markers for these parasites is beyond the scope of the current investigation, this study contributes the first comprehensive knowledge on genome-wide features and 1–10 bp unit motif microsatellite diversity for *P. malariae* and *P. ovale curtisi*. Utilization of these computational outcomes could assist in the identification of novel microsatellite markers for haplotype clustering, population differentiation, and linkage disequilibrium, as demonstrated by the success of such *in silico*-based studies in the past for *P. falciparum*, *P. vivax*, and *Leishmania panamensis* [2, 24, 46].

In conclusion, this study presents the first comprehensive categorization of mono- to deca-nucleotide microsatellites in five human malaria-causing *Plasmodium* species. The results indicate high diversity in the CDS and genomic microsatellite distribution across all investigated species of *Plasmodium*. In *P. malariae* and *P. ovale curtisi*, the high density of microsatellite distribution observed warrants further in-depth investigation to identify potential genotyping markers for epidemiological studies.

## Supplementary materials

Supplementary material is available at https://www.parasite-journal.org/10.1051/parasite/2020034/olmSupplementary Tables*Supplementary Table 1*. Categorization of the most frequently repeated microsatellite motifs in genomes of *Plasmodium* species.*Supplementary Table 2*. Diversity and motif length-wise distribution of chromosomal microsatellite in *P. malariae* UG01.*Supplementary Table 3*. Diversity and motif length-wise distribution of chromosomal microsatellite in *P. ovale curtisi*.**Supplementary Figure 1.**
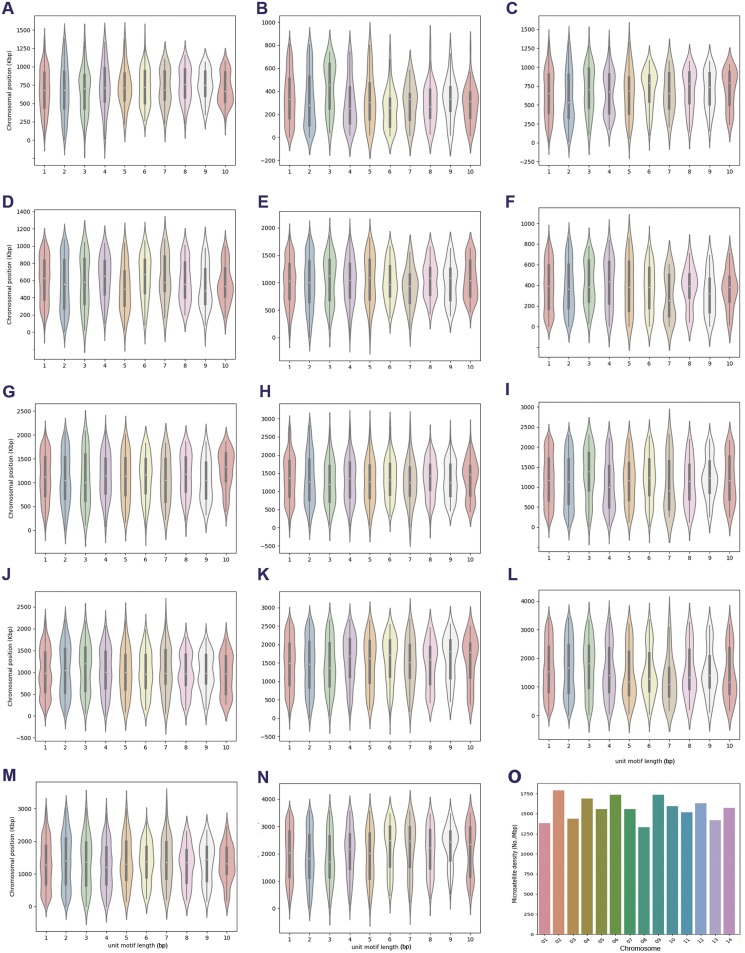
Chromosomal lengthwise distribution of microsatellites based on unit motif length in *Plasmodium malariae* UG01. The subplots (A–N) indicate lengthwise distribution of microsatellites in chromosomes 1–14 separated according to the unit motif length of microsatellites identified for each chromosome. Width of each violin plot indicates relative densities of microsatellites in the region. (O) Summary of microsatellite densities in each chromosome of *P. malariae* UG01.**Supplementary Figure 2.**
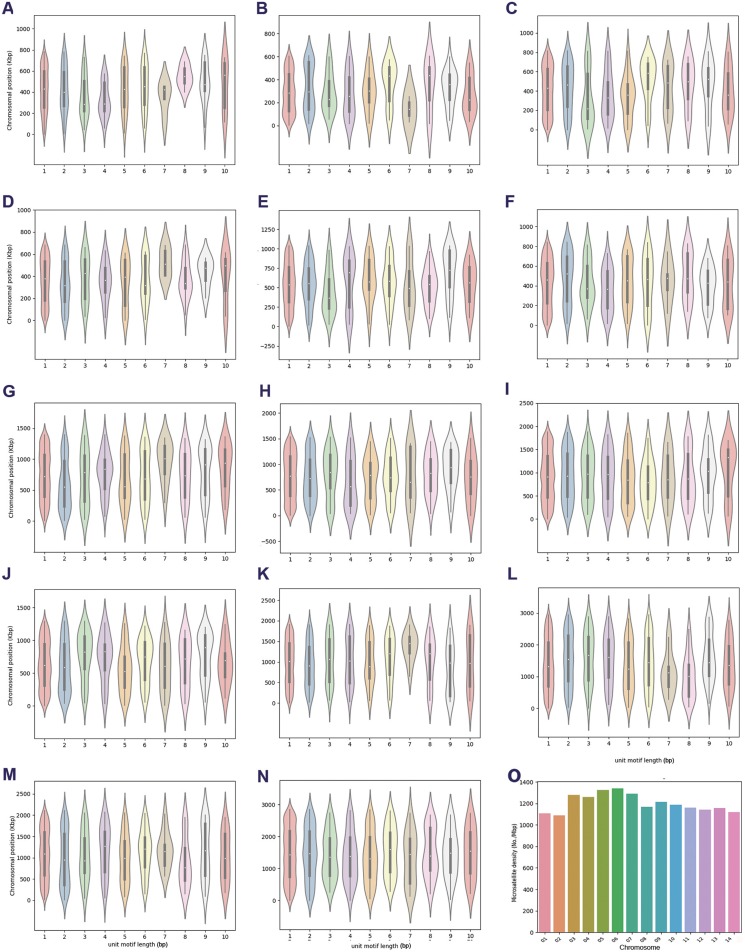
Chromosomal lengthwise distribution of microsatellites based on unit motif length in *Plasmodium ovale curtisi* GH01. The subplots (A–N) indicate lengthwise distribution of microsatellites in chromosomes 1–14 separated according to the unit motif length of microsatellites identified for each chromosome. Width of each violin plot indicates relative densities of microsatellites in the region. (O) Summary of microsatellite densities in each chromosome of *P. ovale curtisi* GH01.**Supplementary Figure 3.**
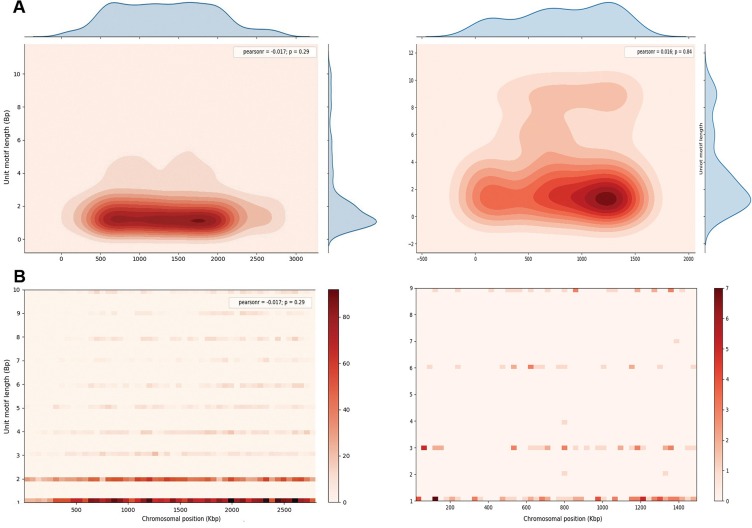
Representative chart for the density of microsatellite distribution in *Plasmodium malariae* UG01 (left) and *P. ovale curtisi* GH01 (right) chromosomes. Overall representation chart of (A) the microsatellite density distribution (B) gradient-wise distribution of the core regions with maximum microsatellite densities for chromosome 8 in *P. malariae*.AbbreviationsAUCarea under curvebpBase pairCDSProtein-coding-regionsGOGene ontologyHEHeterozygosityKbpKilo base pairMbpMillion base pairORFsOpen reading frameSNPSingle nucleotide polymorphisms

## Authors’ contribution statement

VBM and MI designed the study. VBM preformed the data analysis and wrote the first draft of the manuscript. SN assisted in part of data analysis. NJW and AMD assisted in logistic support and manuscript preparation. All authors read and approved the final manuscript.

## Funding

The research was funded by postdoctoral research sponsorship of Mahidol University, Thailand, Thailand Science Research and Innovation (TSRI), grant no. RTA6280006 and the Wellcome Trust of Great Britain, UK.

## Availability of data and materials

The datasets generated during the current study are available from the corresponding author on reasonable request.

## References

[1] Anderson TJ, Su XZ, Bockarie M, Lagog M, Day KP. 1999 Twelve microsatellite markers for characterization of *Plasmodium falciparum* from finger-prick blood samples. Parasitology, 119, 113–125. 10466118 10.1017/s0031182099004552

[2] Anderson TJ, Haubold B, Williams JT, Estrada-Franco JG, Richardson L, Mollinedo R, Bockarie M, Mokili J, Mharakurwa S, French N, Whitworth J, Velez ID, Brockman AH, Nosten F, Ferreira MU, Day KP. 2000 Microsatellite markers reveal a spectrum of population structures in the malaria parasite *Plasmodium falciparum*. Molecular Biology and Evolution, 17, 1467–1482. 11018154 10.1093/oxfordjournals.molbev.a026247

[3] Arnaud MB, Costanzo MC, Shah P, Skrzypek MS, Sherlock G. 2009 Gene Ontology and the annotation of pathogen genomes: the case of *Candida albicans*. Trends in Microbiology, 17(7), 295–303. 19577928 10.1016/j.tim.2009.04.007 PMC3907193

[4] Aurrecoechea C, Brestelli J, Brunk BP, Dommer J, Fischer S, Gajria B, Gao X, Gingle A, Grant G, Harb OS, Heiges M, Innamorato F, Iodice J, Kissinger JC, Kraemer E, Li W, Miller JA, Nayak V, Pennington C, Pinney DF, Roos DS, Ross C, Stoeckert CJ Jr, Treatman C, Wang H. 2009 PlasmoDB: a functional genomic database for malaria parasites. Nucleic Acids Research, 37(Database issue), D539–D543. 18957442 10.1093/nar/gkn814 PMC2686598

[5] Bethke L, Thomas S, Walker K, Lakhia R, Rangarajan R, Wirth D. 2007 The role of DNA mismatch repair in generating genetic diversity and drug resistance in malaria parasites. Molecular and Biochemical Parasitology, 155(1), 18–25. 17583362 10.1016/j.molbiopara.2007.05.003 PMC3683857

[6] Brehelin L, Dufayard JF, Gascuel O. 2008 PlasmoDraft: a database of *Plasmodium falciparum* gene function predictions based on postgenomic data. BMC Bioinformatics, 9, 440. 18925948 10.1186/1471-2105-9-440 PMC2605471

[7] Castagnone-Sereno P, Danchin EG, Deleury E, Guillemaud T, Malausa T, Abad P. 2010 Genome-wide survey and analysis of microsatellites in nematodes, with a focus on the plant-parasitic species *Meloidogyne incognita*. BMC Genomics, 11, 598. 20973953 10.1186/1471-2164-11-598 PMC3091743

[8] Chen M, Zeng G, Tan Z, Jiang M, Zhang J, Zhang C, Lu L, Lin Y, Peng J. 2011 Compound microsatellites in complete *Escherichia coli* genomes. FEBS Letters, 585, 1072–1076. 21382371 10.1016/j.febslet.2011.03.005

[9] Cochrane AH, Collins WE, Nussenzweig RS. 1984 Monoclonal antibody identifies circumsporozoite protein of *Plasmodium malariae* and detects a common epitope on *Plasmodium brasilianum* sporozoites. Infection and Immunity, 45(3), 592–595. 6381308 10.1128/iai.45.3.592-595.1984 PMC263335

[10] Dallas JF. 1992 Estimation of microsatellite mutation rates in recombinant inbred strains of mouse. Mammalian Genome, 3, 452–456. 1643307 10.1007/BF00356155

[11] Davies HM, Thalassinos K, Osborne AR. 2016 Expansion of lysine-rich repeats in *Plasmodium* proteins generates novel localization sequences that target the periphery of the host erythrocyte. Journal of Biological Chemistry, 291, 26,188–26,207. 10.1074/jbc.M116.761213 PMC5207086 27777305

[12] Douglas NM, Lampah DA, Kenangalem E, Simpson JA, Poespoprodjo JR, Sugiarto P, Anstey NM, Price RN. 2013 Major burden of severe anemia from non-falciparum malaria species in Southern Papua: a hospital-based surveillance study. PLoS Medicine, 10, e1001575. 24358031 10.1371/journal.pmed.1001575 PMC3866090

[13] Ellegren H. 2004 Microsatellites: simple sequences with complex evolution. Nature Reviews Genetics, 5, 435–445. 10.1038/nrg1348 15153996

[14] Fan H, Chu JY. 2007 A brief review of short tandem repeat mutation. Genomics, Proteomics & Bioinformatics, 5, 7–14. 10.1016/S1672-0229(07)60009-6 PMC5054066 17572359

[15] Felten A, Vila Nova M, Durimel K, Guillier L, Mistou MY, Radomski N. 2017 First gene-ontology enrichment analysis based on bacterial coregenome variants: insights into adaptations of Salmonella serovars to mammalian- and avian-hosts. BMC Microbiology, 17(1), 222. 29183286 10.1186/s12866-017-1132-1 PMC5706153

[16] Figan CE, Sa JM, Mu J, Melendez-Muniz VA, Liu CH, Wellems TE. 2018 A set of microsatellite markers to differentiate *Plasmodium falciparum* progeny of four genetic crosses. Malaria Journal, 17(1), 60. 29394891 10.1186/s12936-018-2210-z PMC5797376

[17] Gilles HM, Hendrickse RG. 1963 Nephrosis in Nigerian children. Role of *Plasmodium malariae*, and effect of antimalarial treatment. British Medical Journal, 2, 27–31. 13947894 10.1136/bmj.2.5348.27 PMC1872141

[18] Gillings MR, Westoby M. 2014 DNA technology and evolution of the Central Dogma. Trends in Ecology & Evolution, 29, 1–2. 24148293 10.1016/j.tree.2013.10.001

[19] Gomez JC, McNamara DT, Bockarie MJ, Baird JK, Carlton JM, Zimmerman PA. 2003 Identification of a polymorphic *Plasmodium vivax* microsatellite marker. American Journal of Tropical Medicine and Hygiene, 69(4), 377–379. 14640496 PMC3728893

[20] Greenhouse B, Myrick A, Dokomajilar C, Woo JM, Carlson EJ, Rosenthal PJ, Dorsey G. 2006 Validation of microsatellite markers for use in genotyping polyclonal *Plasmodium falciparum* infections. American Journal of Tropical Medicine and Hygiene, 75(5), 836–842. 17123974 PMC1697796

[21] Groger M, Veletzky L, Lalremruata A, Cattaneo C, Mischlinger J, Manego Zoleko R, Kim J, Klicpera A, Meyer EL, Blessborn D, Winterberg M, Adegnika AA, Agnandji ST, Kremsner PG, Mordmuller B, Mombo-Ngoma G, Fuehrer HP, Ramharter M. 2019 Prospective clinical and molecular evaluation of potential *Plasmodium ovale curtisi* and *wallikeri* relapses in a high-transmission setting. Clinical Infectious Diseases, 69(12), 2119–2126. 31066448 10.1093/cid/ciz131 PMC6880329

[22] Guichoux E, Lagache L, Wagner S, Chaumeil P, Leger P, Lepais O, Lepoittevin C, Malausa T, Revardel E, Salin F, Petit RJ. 2011 Current trends in microsatellite genotyping. Molecular Ecology Resources, 11, 591–611. 21565126 10.1111/j.1755-0998.2011.03014.x

[23] Hamilton WL, Claessens A, Otto TD, Kekre M, Fairhurst RM, Rayner JC, Kwiatkowski D. 2017 Extreme mutation bias and high AT content in *Plasmodium falciparum*. Nucleic Acids Research, 45, 1889–1901. 27994033 10.1093/nar/gkw1259 PMC5389722

[24] Imwong M, Nair S, Pukrittayakamee S, Sudimack D, Williams JT, Mayxay M, Newton PN, Kim JR, Nandy A, Osorio L, Carlton JM, White NJ, Day NP, Anderson TJ. 2007 Contrasting genetic structure in *Plasmodium vivax* populations from Asia and South America. International Journal for Parasitology, 37, 1013–1022. 17442318 10.1016/j.ijpara.2007.02.010

[25] Jarne P, Lagoda PJ. 1996 Microsatellites, from molecules to populations and back. Trends in Ecology & Evolution, 11, 424–429. 21237902 10.1016/0169-5347(96)10049-5

[26] Katti MV, Sami-Subbu R, Ranjekar PK, Gupta VS. 2000 Amino acid repeat patterns in protein sequences: their diversity and structural-functional implications. Protein Science, 9, 1203–1209. 10892812 10.1110/ps.9.6.1203 PMC2144659

[27] Koepfli C, Mueller I, Marfurt J, Goroti M, Sie A, Oa O, Genton B, Beck HP, Felger I. 2009 Evaluation of *Plasmodium vivax* genotyping markers for molecular monitoring in clinical trials. Journal of Infectious Diseases, 199(7), 1074–1080. 19275476 10.1086/597303

[28] Lai Y, Sun F. 2003 The relationship between microsatellite slippage mutation rate and the number of repeat units. Molecular Biology and Evolution, 20, 2123–2131. 12949124 10.1093/molbev/msg228

[29] Langford S, Douglas NM, Lampah DA, Simpson JA, Kenangalem E, Sugiarto P, Anstey NM, Poespoprodjo JR, Price RN. 2015 *Plasmodium malariae* infection associated with a high burden of anemia: a hospital-based surveillance study. PLoS Neglected Tropical Diseases, 9, e0004195. 26720002 10.1371/journal.pntd.0004195 PMC4697806

[30] Levinson G, Gutman GA. 1987 High frequencies of short frameshifts in poly-CA/TG tandem repeats borne by bacteriophage M13 in *Escherichia coli* K-12. Nucleic Acids Research, 15, 5323–5338. 3299269 10.1093/nar/15.13.5323 PMC305964

[31] Liu Y, Zhou RM, Zhang YL, Wang DQ, Li SH, Yang CY, Qian D, Zhao YL, Zhang HW, Xu BL. 2018 Analysis of polymorphisms in the circumsporozoite protein gene of *Plasmodium vivax* isolates from Henan Province, China. Malaria Journal, 17(1), 103. 29506527 10.1186/s12936-018-2237-1 PMC5838951

[32] Lo E, Nguyen K, Nguyen J, Hemming-Schroeder E, Xu J, Etemesi H, Githeko A, Yan G. 2017 *Plasmodium malariae* prevalence and csp gene diversity, Kenya, 2014 and 2015. Emerging Infectious Diseases, 23(4), 601–610. 28322694 10.3201/eid2304.161245 PMC5367407

[33] Lover AA, Baird JK, Gosling R, Price RN. 2018 Malaria elimination: time to target all species. American Journal of Tropical Medicine and Hygiene, 99(1), 17–23. 29761762 10.4269/ajtmh.17-0869 PMC6035869

[34] Mathema VB, Dondorp AM, Imwong M. 2019 OSTRFPD: Multifunctional tool for genome-wide short tandem repeat analysis for DNA, transcripts, and amino acid sequences with integrated primer designer. Evolutionary Bioinformatics Online, 15, 1176934319843130. 31040636 10.1177/1176934319843130 PMC6482647

[35] Mayer C. 2010 Phobos – a tandem repeat search tool for complete genomes. http://www.ruhr-uni-bochum.de/spezzoo/cm. Accessed on 25 April 2018.

[36] Mendes TA, Lobo FP, Rodrigues TS, Rodrigues-Luiz GF, daRocha WD, Fujiwara RT, Teixeira SM, Bartholomeu DC. 2013 Repeat-enriched proteins are related to host cell invasion and immune evasion in parasitic protozoa. Molecular Biology and Evolution, 30, 951–963. 23303306 10.1093/molbev/mst001

[37] Menegon M, Bardaji A, Martinez-Espinosa F, Botto-Menezes C, Ome-Kaius M, Mueller I, Betuela I, Arevalo-Herrera M, Kochar S, Kochar SK, Jaju P, Hans D, Chitnis C, Padilla N, Castellanos ME, Ortiz L, Sanz S, Piqueras M, Desai M, Mayor A, Del Portillo H, Menendez C, Severini C. 2016 Microsatellite genotyping of *Plasmodium vivax* isolates from pregnant women in four malaria endemic countries. PloS One, 11(3), e0152447. 27011010 10.1371/journal.pone.0152447 PMC4807005

[38] Metzgar D, Bytof J, Wills C. 2000 Selection against frameshift mutations limits microsatellite expansion in coding DNA. Genome Research, 10, 72–80. 10645952 PMC310501

[39] Milner DA Jr. 2018 Malaria pathogenesis. Cold Spring Harbor Perspectives in Medicine, 8(1), a025569. 28533315 10.1101/cshperspect.a025569 PMC5749143

[40] Mueller I, Zimmerman PA, Reeder JC. 2007 *Plasmodium malariae* and *Plasmodium ovale* – the “bashful” malaria parasites. Trends in Parasitology, 23(6), 278–283. 17459775 10.1016/j.pt.2007.04.009 PMC3728836

[41] Nino CH, Cubides JR, Camargo-Ayala PA, Rodriguez-Celis CA, Quinones T, Cortes-Castillo MT, Sanchez-Suarez L, Sanchez R, Patarroyo ME, Patarroyo MA. 2016 *Plasmodium malariae* in the Colombian Amazon region: you don’t diagnose what you don’t suspect. Malaria Journal, 15, 576. 27899111 10.1186/s12936-016-1629-3 PMC5129613

[42] Nojadeh JN, Behrouz Sharif S, Sakhinia E. 2018 Microsatellite instability in colorectal cancer. EXCLI Journal, 17, 159–168. 29743854 10.17179/excli2017-948 PMC5938532

[43] Oguike MC, Betson M, Burke M, Nolder D, Stothard JR, Kleinschmidt I, Proietti C, Bousema T, Ndounga M, Tanabe K, Ntege E, Culleton R, Sutherland CJ. 2011 *Plasmodium ovale curtisi* and *Plasmodium ovale wallikeri* circulate simultaneously in African communities. International Journal for Parasitology, 41(6), 677–683. 21315074 10.1016/j.ijpara.2011.01.004 PMC3084460

[44] Olson M, Hood L, Cantor C, Botstein D. 1989 A common language for physical mapping of the human genome. Science, 245(4925), 1434–1435. 2781285 10.1126/science.2781285

[45] Orjuela-Sanchez P, Sa JM, Brandi MC, Rodrigues PT, Bastos MS, Amaratunga C, Duong S, Fairhurst RM, Ferreira MU. 2013 Higher microsatellite diversity in *Plasmodium vivax* than in sympatric *Plasmodium falciparum* populations in Pursat, Western Cambodia. Experimental Parasitology, 134(3), 318–326. 23562882 10.1016/j.exppara.2013.03.029 PMC3691688

[46] Oyebola MK, Idowu ET, Nyang H, Olukosi YA, Otubanjo OA, Nwakanma DC, Awolola ST, Amambua-Ngwa A. 2014 Microsatellite markers reveal low levels of population sub-structuring of *Plasmodium falciparum* in southwestern Nigeria. Malaria Journal, 13, 493. 25496185 10.1186/1475-2875-13-493 PMC4300683

[47] Roman DNR, Rosalie NNA, Kumar A, Luther KMM, Singh V, Albert MS. 2018 Asymptomatic *Plasmodium malariae* infections in children from suburban areas of Yaounde, Cameroon. Parasitology International, 67(1), 29–33. 28263883 10.1016/j.parint.2017.02.009

[48] Roucher C, Rogier C, Sokhna C, Tall A, Trape JF. 2014 A 20-year longitudinal study of *Plasmodium ovale* and *Plasmodium malariae* prevalence and morbidity in a West African population. PloS One, 9(2), e87169. 24520325 10.1371/journal.pone.0087169 PMC3919715

[49] Rutledge GG, Bohme U, Sanders M, Reid AJ, Cotton JA, Maiga-Ascofare O, Djimde AA, Apinjoh TO, Amenga-Etego L, Manske M, Barnwell JW, Renaud F, Ollomo B, Prugnolle F, Anstey NM, Auburn S, Price RN, McCarthy JS, Kwiatkowski DP, Newbold CI, Berriman M, Otto TD. 2017 *Plasmodium malariae* and *P. ovale* genomes provide insights into malaria parasite evolution. Nature, 542, 101–104. 28117441 10.1038/nature21038 PMC5326575

[50] Rutledge GG, Marr I, Huang GKL, Auburn S, Marfurt J, Sanders M, White NJ, Berriman M, Newbold CI, Anstey NM, Otto TD, Price RN. 2017 Genomic characterization of recrudescent *Plasmodium malariae* after treatment with artemether/lumefantrine. Emerging Infectious Diseases, 23, 1300–1307. 28430103 10.3201/eid2308.161582 PMC5547787

[51] Saralamba N, Mayxay M, Newton PN, Smithuis F, Nosten F, Archasuksan L, Pukrittayakamee S, White NJ, Day NPJ, Dondorp AM, Imwong M. 2018 Genetic polymorphisms in the circumsporozoite protein of *Plasmodium malariae* show a geographical bias. Malaria Journal, 17(1), 269. 30012172 10.1186/s12936-018-2413-3 PMC6048912

[52] Schindel DE, Miller SE. 2005 DNA barcoding a useful tool for taxonomists. Nature, 435, 17. 10.1038/435017b 15874991

[53] Schindler T, Robaina T, Sax J, Bieri JR, Mpina M, Gondwe L, Acuche L, Garcia G, Cortes C, Maas C, Daubenberger C. 2019 Molecular monitoring of the diversity of human pathogenic malaria species in blood donations on Bioko Island, Equatorial Guinea. Malaria Journal, 18(1), 9. 30646918 10.1186/s12936-019-2639-8 PMC6332537

[54] Selkoe KA, Toonen RJ. 2006 Microsatellites for ecologists: a practical guide to using and evaluating microsatellite markers. Ecology Letters, 9, 615–629. 16643306 10.1111/j.1461-0248.2006.00889.x

[55] Sharma PC, Grover A, Kahl G. 2007 Mining microsatellites in eukaryotic genomes. Trends in Biotechnology, 25, 490–498. 17945369 10.1016/j.tibtech.2007.07.013

[56] Simbaqueba J, Sanchez P, Sanchez E, Nunez Zarantes VM, Chacon MI, Barrero LS, Marino-Ramirez L. 2011 Development and characterization of microsatellite markers for the Cape gooseberry *Physalis peruviana*. PloS One, 6, e26719. 22039540 10.1371/journal.pone.0026719 PMC3198794

[57] Soontarawirat I, Andolina C, Paul R, Day NPJ, Nosten F, Woodrow CJ, Imwong M. 2017 *Plasmodium vivax* genetic diversity and heterozygosity in blood samples and resulting oocysts at the Thai-Myanmar border. Malaria Journal, 16(1), 355. 28870214 10.1186/s12936-017-2002-x PMC5584506

[58] Szalkai B, Grolmusz V. 2018 SECLAF: a webserver and deep neural network design tool for hierarchical biological sequence classification. Bioinformatics, 34, 2487–2489. 29490010 10.1093/bioinformatics/bty116

[59] Tan JC, Tan A, Checkley L, Honsa CM, Ferdig MT. 2010 Variable numbers of tandem repeats in *Plasmodium falciparum* genes. Journal of Molecular Evolution, 71, 268–278. 20730584 10.1007/s00239-010-9381-8 PMC3205454

[60] Toth G, Gaspari Z, Jurka J. 2000 Microsatellites in different eukaryotic genomes: survey and analysis. Genome Research, 10, 967–981. 10899146 10.1101/gr.10.7.967 PMC310925

[61] Trevino SG, Nkhoma SC, Nair S, Daniel BJ, Moncada K, Khoswe S, Banda RL, Nosten F, Cheeseman IH. 2017 High-resolution single-cell sequencing of malaria parasites. Genome Biology and Evolution, 9, 3373–3383. 29220419 10.1093/gbe/evx256 PMC5737330

[62] Trimarsanto H, Benavente ED, Noviyanti R, Utami RA, Trianty L, Pava Z, Getachew S, Kim JY, Goo YK, Wangchuck S, Liu Y, Gao Q, Dowd S, Cheng Q, Clark TG, Price RN, Auburn S. 2017 VivaxGEN: an open access platform for comparative analysis of short tandem repeat genotyping data in *Plasmodium vivax* populations. PLoS Neglected Tropical Diseases, 11, e0005465. 28362818 10.1371/journal.pntd.0005465 PMC5389845

[63] Tripura R, Peto TJ, Chea N, Chan D, Mukaka M, Sirithiranont P, Dhorda M, Promnarate C, Imwong M, von Seidlein L, Duanguppama J, Patumrat K, Huy R, Grobusch MP, Day NPJ, White NJ, Dondorp AM. 2018 A controlled trial of mass drug administration to interrupt transmission of multidrug-resistant falciparum malaria in Cambodian villages. Clinical Infectious Diseases, 67(6), 817–826. 29522113 10.1093/cid/ciy196 PMC6117448

[64] Vieira ML, Santini L, Diniz AL, Munhoz Cde F. 2016 Microsatellite markers: what they mean and why they are so useful. Genetics and Molecular Biology, 39, 312–328. 27561112 10.1590/1678-4685-GMB-2016-0027 PMC5004837

[65] Weber JL, Wong C. 1993 Mutation of human short tandem repeats. Human Molecular Genetics, 2, 1123–1128. 8401493 10.1093/hmg/2.8.1123

[66] WHO. 2016 Eliminating malaria in the Greater Mekong Subregion: united to end a deadly disease. p. 24.

[67] Yamamoto H, Imai K. 2015 Microsatellite instability: an update. Archives of Toxicology, 89(6), 899–921. 25701956 10.1007/s00204-015-1474-0

[68] Yman V, Wandell G, Mutemi DD, Miglar A, Asghar M, Hammar U, Karlsson M, Lind I, Nordfjell C, Rooth I, Ngasala B, Homann MV, Farnert A. 2019 Persistent transmission of *Plasmodium malariae* and *Plasmodium ovale* species in an area of declining *Plasmodium falciparum* transmission in eastern Tanzania. PLoS Neglected Tropical Diseases, 13(5), e0007414.31136585

[69] Zhou M, Liu Q, Wongsrichanalai C, Suwonkerd W, Panart K, Prajakwong S, Pensiri A, Kimura M, Matsuoka H, Ferreira MU, Isomura S, Kawamoto F. 1998 High prevalence of *Plasmodium malariae* and *Plasmodium ovale* in malaria patients along the Thai-Myanmar border, as revealed by acridine orange staining and PCR-based diagnoses. Tropical Medicine & International Health, 3(4), 304–312. 9623932 10.1046/j.1365-3156.1998.00223.x

