# Supplementary material for: Genome-wide microsatellite characteristics of five human Plasmodium species, focusing on Plasmodium malariae and P. ovale curtisi
Source: Parasite. 2020 May 15;27:34. doi: 10.1051/parasite/2020034 (PMC7227371; doi:10.1051/parasite/2020034)
Supplement: Supplementary Tables — Supplementary Table 1. Categorization of the most frequently repeated microsatellite motifs in genomes of Plasmodium species. Supplementary Table 2. Diversity and motif length-wise distribution of chromosomal microsatellite in P. malariae UG01. Supplementary Table 3. Diversity and motif length-wise distribution of chromosomal microsatellite in P. ovale curtisi. [file parasite-27-34-s1.pdf]

Supplementary Materials

Supplementary Table 1. Categorization of the most frequently repeated microsatellite motifs in genomes of *Plasmodium* species

***Plasmodium* species**	**Unit motif length (bp)[a]**									
	**1**	**2**	**3**	**4**	**5**	**6**	**7**	**8**	**9**	**10**
P. falciparum 3D7	A(27682)	AT(35293)	AAT(5932)	AAAT(5384)	AAAAT(1715)	AAAAAT(417)	AAAAAAT(312)	AAATATAT(317)	AATATATAT(176)	AAATATATAT(119)
	C(<10)	AC(93)	ATC(389)	ACAT(506)	AATAT(710)	AAATAT(312)	AATATAT(256)	AAAAAAAT(191)	AAAAAAAAT(136)	AAAAAAAAAT(69)
		AG(37)	AAG(244)	AAAG(132)	AAAAG(178)	AAAAAG(228)	AAAAAAG(104)	ACATATAT(115)	AATAATATG(60)	ACATATATAT(54)
			AAC(183)	AAAC(79)	AAAAC(71)	AATGAT(64)	AAATAAT(55)	AAAAAAAG(72)	AAAAAAAAG(52)	AAATAAATAT(31)
P. vivax SAL-1	A(15404)	AT(633)	AAG(188)	AAAT(206)	AAAAT(199)	AAAAAG(59)	AAACCCT(58)	AAAAAAAT(34)	AAAAAAAAG(19)	AAAAAAAAAG(26)
	C(140)	AC(176)	AGG(116)	ACAT(101)	AAAAG(55)	AAAAAT(43)	AAAAAAT(47)	AAGCGGTG(21)	AACACCGCT(12)	AAAAAAAAGG(16)
		AG(37)	AAT(95)	AAAC(46)	AAAAC(31)	ACATAT(29)	AAAAAAG(19)	ACATATAT(19)	AAAAAAAAT(12)	AAAAGGAATT(<10)
			ATC(58)	AAAG(45)	AAAGC(24)	AAAAGG(24)	AAAATGC(<10)	AAAAAAAG(19)	AAAAAAAGG(11)	AAAAAAAAAC(<10)
P. malariae UG01	A(24319)	AT(11597)	AAT(1285)	ACAT(1094)	AAAAT(1018)	ACATAT(214)	AAAAAAT(110)	ACATATAT(177)	AAAAAAAAT(43)	ACATATATAT(89)
	C(1)	AC(222)	AAG(188)	AAAT(842)	AAAAG(154)	AAAAAT(238)	AAAAAAG(100)	AAAAAAAT(77)	AAAAAAAAG(41)	ACATACATAT(54)
		AG(38)	ACT(176)	AAAG(82)	AAAAC(103)	AAAAAG(196)	AATATAT(36)	AAAAAAAG(64)	AATAATAGT(17)	AAAAAAAAAT(40)
			ATC(113)	AAAC(76)	AATAT(78)	AATAGT(94)	AAATAAT(30)	AAAATAAT(54)	AAAATAAAT(15)	AAAAAAAAAG(35)
P. ovale curtisi GH01	A(20859)	AT(3413)	AAT(404)	ACAT(266)	AAAAT(526)	AAAAAT(91)	AAAAAAT(38)	ACATATAT(35)	AAAAAAAAG(4)	ACATATATAT(11)
	C(126)	AC(214)	AAG(230)	AAAT(256)	AAAAG(90)	AAAAAG(76)	AAAAAAG(33)	AAAAAAAT(28)	AACACTGCT(3)	AAAAAAAAAT(11)
		AG(94)	ATC(55)	AAAG(49)	AAAAC(44)	ACATAT(66)	AAAAAGG(16)	AAAAAAAG(22)	AAGAAGAGG(3)	ACATACATAT(<10)
			AAC(29)	AAAC(27)	AATAT(41)	AAAAGG(25)	AAAAATG(<10)	AAAAAAAAG(14)	AATATATAT(3)	ACATATATAT(<10)
P. knowlesi STRAIN-H	A(23592)	AT(2308)	AAT(247)	AAAT(268)	AAAAT(393)	ACATAT(205)	AAACCCT(364)	ACATATAT(268)	AACACAACC(63)	ACATATATAT(102)
	C(215)	AC(307)	AAG(203)	ACAT(159)	AAAAG(176)	ATATGC(135)	AAAAAAG(79)	AACATACT(59)	AACATAACC(62)	AAAAAAAAAG(25)
		AG(27)	ATC(102)	AAAG(70)	AAAGG(71)	AAAAGG(50)	AACCCTG(65)	AAAAAAAG(57)	AAAAAAAAG(19)	ACATACATAT(15)
			AAC(74)	AAAC(48)	AACAC(58)	AAAAAT(48)	AAAAAAT(50)	AAAAGAAG(36)	AAAAAAAAG(43)	AAAGGGAAGG(13)

[a] Entire genomes of five *Plasmodium* species causing human malaria were scanned for diversity of microsatellite 1-10 bp unit motif length. List contains four most frequently repeated unique motifs along with their occurrence for each unit motif length.

Supplementary Table 2. Diversity and motif length-wise distribution of chromosomal microsatellite in *P. malariae* UG01

	Unit motif length (n)[a]									
	**1**	**2**	**3**	**4**	**5**	**6**	**7**	**8**	**9**	**10**
Chr-1	990 (51.86%)	442 (23.15%)	85 (4.45%)	77 (4.03%)	53 (2.78%)	69 (3.61%)	30 (1.57%)	58 (3.04%)	47 (2.46%)	58 (3.04%)
Chr-2	758 (52.06%)	327 (22.46%)	51 (3.5%)	62 (4.26%)	54 (3.71%)	57 (3.91%)	25 (1.72%)	42 (2.88%)	30 (2.06%)	50 (3.43%)
Chr-3	978 (50.65%)	414 (21.44%)	59 (3.06%)	113 (5.85%)	81 (4.19%)	95 (4.92%)	16 (.83%)	68 (3.52%)	56 (2.9%)	51 (2.64%)
Chr-4	914 (50.3%)	418 (23.00%)	74 (4.07%)	71 (3.91%)	50 (2.75%)	71 (3.91%)	28 (1.54%)	86 (4.73%)	33 (1.82%)	72 (3.96%)
Chr-5	1447 (49.18%)	749 (25.46%)	105 (3.57%)	147 (5.00%)	97 (3.3%)	101 (3.43%)	40 (1.36%)	93 (3.16%)	80 (2.72%)	83 (2.82%)
Chr-6	755 (50.4%)	327 (21.83%)	70 (4.67%)	76 (5.07%)	59 (3.94%)	64 (4.27%)	24 (1.6%)	39 (2.6%)	38 (2.54%)	46 (3.07%)
Chr-7	1666 (51.42%)	766 (23.64%)	105 (3.24%)	158 (4.88%)	103 (3.18%)	97 (2.99%)	55 (1.7%)	112 (3.46%)	67 (2.07%)	111 (3.43%)
Chr-8	1892 (50.37%)	931 (24.79%)	153 (4.07%)	173 (4.61%)	130 (3.46%)	127 (3.38%)	41 (1.09%)	109 (2.9%)	96 (2.56%)	104 (2.77%)
Chr-9	2028 (50.52%)	959 (23.89%)	171 (4.26%)	186 (4.63%)	116 (2.89%)	180 (4.48%)	41 (1.02%)	106 (2.64%)	122 (3.04%)	105 (2.62%)
Chr-10	1849 (52.23%)	849 (23.98%)	125 (3.53%)	167 (4.72%)	99 (2.8%)	116 (3.28%)	47 (1.33%)	122 (3.45%)	83 (2.34%)	83 (2.34%)
Chr-11	2075 (50.28%)	1023 (24.79%)	190 (4.6%)	220 (5.33%)	133 (3.22%)	159 (3.85%)	52 (1.26%)	97 (2.35%)	94 (2.28%)	84 (2.04%)
Chr-12	2908 (51.89%)	1383 (24.68%)	241 (4.3%)	271 (4.84%)	174 (3.1%)	204 (3.64%)	64 (1.14%)	138 (2.46%)	99 (1.77%)	122 (2.18%)
Chr-13	2207 (51.42%)	1066 (24.84%)	173 (4.03%)	211 (4.92%)	122 (2.84%)	134 (3.12%)	64 (1.49%)	112 (2.61%)	97 (2.26%)	106 (2.47%)
Chr-14	2890 (51.55%)	1424 (25.4%)	229 (4.08%)	270 (4.82%)	172 (3.07%)	154 (2.75%)	75 (1.34%)	150 (2.68%)	104 (1.86%)	138 (2.46%)

[a] Entire chromosomal DNA of *Plasmodium malariae* UG01 was scanned for microsatellites and categorized according to unit motif length. Data are expressed as motif-wise number of microsatellites and corresponding percentage within each chromosome.

Supplementary Table 3. Diversity and motif length-wise distribution of chromosomal microsatellite in *P. ovale curtisi*

Unit motif length (n)[a]

	1	2	3	4	5	6	7	8	9	10
**Chr-1**	651 (74.91%)	86 (9.9%)	25 (2.88%)	16 (1.84%)	25 (2.88%)	26 (2.99%)	4 (0.46%)	7 (0.81%)	22 (2.53%)	7 (0.81%)
**Chr-2**	498 (75.23%)	61 (9.21%)	14 (2.11%)	18 (2.72%)	14 (2.11%)	18 (2.72%)	2 (0.3%)	8 (1.21%)	17 (2.57%)	12 (1.81%)
**Chr-3**	810 (77.14%)	81 (7.71%)	24 (2.29%)	28 (2.67%)	27 (2.57%)	28 (2.67%)	12 (1.14%)	14 (1.33%)	15 (1.43%)	11 (1.05%)
**Chr-4**	663 (76.91%)	79 (9.16%)	16 (1.86%)	20 (2.32%)	28 (3.25%)	19 (2.2%)	4 (0.46%)	18 (2.09%)	8 (0.93%)	7 (0.81%)
**Chr-5**	1046 (76.02%)	126 (9.16%)	26 (1.89%)	25 (1.82%)	25 (1.82%)	39 (2.83%)	16 (1.16%)	32 (2.33%)	25 (1.82%)	16 (1.16%)
**Chr-6**	852 (75.67%)	99 (8.79%)	29 (2.58%)	24 (2.13%)	28 (2.49%)	32 (2.84%)	5 (0.44%)	17 (1.51%)	23 (2.04%)	17 (1.51%)
**Chr-7**	1423 (78.84%)	118 (6.54%)	42 (2.33%)	34 (1.88%)	53 (2.94%)	43 (2.38%)	13 (0.72%)	24 (1.33%)	30 (1.66%)	25 (1.39%)
**Chr-8**	1379 (76.95%)	147 (8.2%)	50 (2.79%)	31 (1.73%)	52 (2.9%)	45 (2.51%)	12 (0.67%)	29 (1.62%)	29 (1.62%)	18 (1.%)
**Chr-9**	1766 (77.29%)	186 (8.14%)	50 (2.19%)	43 (1.88%)	73 (3.19%)	64 (2.8%)	13 (0.57%)	33 (1.44%)	37 (1.62%)	20 (0.88%)
**Chr-10**	1154 (74.84%)	121 (7.85%)	49 (3.18%)	27 (1.75%)	53 (3.44%)	45 (2.92%)	22 (1.43%)	21 (1.36%)	34 (2.2%)	16 (1.04%)
**Chr-11**	1660 (74.77%)	206 (9.28%)	50 (2.25%)	55 (2.48%)	65 (2.93%)	77 (3.47%)	11 (0.5%)	34 (1.53%)	41 (1.85%)	21 (0.95%)
**Chr-12**	2541 (75.49%)	311 (9.24%)	69 (2.05%)	73 (2.17%)	96 (2.85%)	112 (3.33%)	25 (0.74%)	35 (1.04%)	63 (1.87%)	41 (1.22%)
**Chr-13**	1873 (76.67%)	214 (8.76%)	48 (1.96%)	40 (1.64%)	75 (3.07%)	86 (3.52%)	13 (0.53%)	33 (1.35%)	35 (1.43%)	26 (1.06%)
**Chr-14**	2399 (75.02%)	322 (10.07%)	79 (2.47%)	62 (1.94%)	93 (2.91%)	86 (2.69%)	27 (0.84%)	42 (1.31%)	53 (1.66%)	35 (1.09%)

[a] Entire chromosomal DNA of *Plasmodium ovale curtisi* was scanned for microsatellites and categorized according to unit motif length. Data are expressed as motif-wise number of microsatellites and corresponding percentage within each chromosome.
